# LncRNA UCA1 promotes cisplatin resistance in gastric cancer via recruiting EZH2 and activating PI3K/AKT pathway

**DOI:** 10.7150/jca.43446

**Published:** 2020-04-06

**Authors:** Qingqiang Dai, Tianqi Zhang, Jiaomeng Pan, Chen Li

**Affiliations:** 1 Department of Surgery, Shanghai Key Laboratory of Gastric Neoplasms, Shanghai Institute of Digestive Surgery, Ruijin Hospital, Shanghai Jiao Tong University School of Medicine, Shanghai 200025, People's Republic of China.; 2 Department of Liver Surgery and Transplantation, Zhongshan Hospital, Fudan University, Shanghai, 200032 China.

**Keywords:** cisplatin resistance, EZH2, gastric cancer, PI3K/AKT pathway, UCA1

## Abstract

**Background**: Drug resistance of cancer cells is one of the major causes of chemotherapy failure. Recently research demonstrated that long non-coding RNA Urothelial cancer associated 1 (UCA1) could promote tumor cisplatin resistance. In this study, we aim to investigate the role of UCA1 in the cisplatin treatment of gastric cancer and its underlying mechanism.

**Methods**: Cell counting kit-8 (CCK-8) assay and apoptosis assay were used to detect the effects of different doses of cisplatin on the proliferation and apoptosis of gastric cancer. We examined the expression relationship between the Enhancer of Zeste Homologue 2 (EZH2) and UCA1 by quantitative Real-time polymerase chain reaction (qRT-PCR) and western blot analysis. Western blot analysis was also performed to detect the expression levels of apoptosis-related proteins, EZH2 and key genes in PI3K/AKT signaling pathway, RIP and RNA pull down assays were performed to explore the interaction between UCA1 and EZH2.

**Results**: We demonstrated that higher the UCA1 expression levels in GC tissues correlated with the poorer the prognosis of patients according to the TCGA database, the GEO database. Moreover, overexpression of UCA1 promotes GC cell proliferation and inhibits cisplatin-induced apoptosis. Knockdown of UCA1 showed the opposite results. Besides, UCA1 exerted its function through interacting with EZH2 and regulates EZH2 expression, knockdown of EZH2 decreased cisplatin resistance of GC cells. Hence, UCA1 promotes cisplatin resistance of GC via recruiting EZH2 and activating PI3K/AKT pathway.

**Conclusion**: Our research revealed the lncRNA UCA1 promoted the cisplatin resistance of GC by recruiting EZH2 and activating PI3K/AKT pathway to modulate cell apoptosis, indicating treatments targeting UCA1 or EZH2 might provide meaningful therapeutic strategies for cisplatin-resistance GC patients.

## Introduction

Gastric cancer (GC) is one of the most commonly diagnosed cancers and the third leading cause of cancer-induced death worldwide which has caused tremendous burdens throughout the world especially in eastern Asia 1-3. Chemotherapy has been widely applied in clinical treatment of GC in recent years. Cisplatin is the most common chemotherapeutic drug in clinical application. However, some GC patients demonstrate cisplatin chemotherapy failure due to drug resistance. Therefore, cisplatin resistance remains a major limitation in the long-term therapeutic efficacy of GC. However, the molecular mechanisms involved in cisplatin resistance still remain unclear.

Long non-coding RNAs (lncRNAs) are RNAs with a length of more than 200 nucleotides. Recently, researches have shown that a huge number of lncRNAs are differentially expressed in tumors 4-6. LncRNAs are showed to associate with the proliferation, metastasis and apoptosis of various tumor cells through the regulation of different signaling pathways 4-6. The intracellular apoptotic pathway and the anti-apoptotic pathway function contribute to the maintenance of normal cell cycle 7. LncRNAs regulate tumor drug resistance and the apoptosis of tumor cells via the interaction with key apoptosis regulators 8, 9. The caspase family is a protease system that directly causes the decomposition of apoptotic cells, which demonstrates an important role in the network of apoptosis mechanisms 7. Many researches demonstrated that lncRNA UCA1 associates with the cisplatin resistance in a variety of tumors 10-13. LncRNA UCA1, which is capable of controlling the activation of multiple signaling pathways in cancers, has been extensively researched in many tumors, including GC 10-15.

In this research, we demonstrated that UCA1 could promote cisplatin resistance of GC via regulating EZH2 to activate PI3K/AKT pathway to modulate GC cell apoptosis and UCA1 may provide a potential therapeutic target for the clinical intervention of GC.

## Methods

### TCGA and GEO database analysis

GC gene expression data and the clinical information were retrieved from TCGA database. Receiver operating characteristic was plotted and an optimal cut-off value was applied to classify patients into low- and high-expression groups referred to the method elucidated by Xiang 16 according to the expression level of UCA1.For validation, the gene expression profiles of GSE62254 were downloaded from GEO database. GSE62254 dataset which was based on GPL570 platform (Affymetrix Human Genome U133 Plus 2.0 Array) contained 300 gastric cancer patients' samples. The clinical information of the dataset was referred to Cristescu R 17. The survival rate of these two groups was then calculated by the Kaplan‐Meier method and log‐rank test by R with the “survival” package.

### Patients and cell culture

GC tissues and matched normal tissues were collected from 30 patients with GC in Ruijin Hospital between 2018 to 2019. The study was approved by the Human Research Ethics Committee of Ruijin Hospital, Shanghai Jiao Tong University, School of Medicine. GC cell lines, NCI-N87 was purchased from American Type Culture Collection (ATCC). The GC cell lines HGC27, MKN45, MGC803, MKN28, AGS, SGC7901 and the immortalized normal gastric epithelial cell line, GES-1, were purchased from Shanghai Institute for Biological Sciences, Chinese Academy of Sciences. All cell lines were grown in RPMI1640 medium (Gibco, BRL, San Francisco, CA, USA) supplemented with 5 μg/ml penicillin/streptomycin and 10% fetal bovine serum (Invitrogen, Carlsbad, CA, USA) in a humidified incubator at 37 °C with 5% CO_2_.

### Cell transfection

The vector pcDNA-UCA1, UCA1 siRNA and their negative control were kindly granted by Wang 18. EZH2 siRNA sequences and negative control sequences were designed and synthesized by Genomeditech(Genomeditech, Shanghai, China). Sequences of siRNAs were listed in supplementary Table S1. For transfection, the vector pcDNA-UCA1, siUCA1, siEZH2 or control reagent was transfected into cells using Lipofectamine 2000 reagent (Invitrogen) following the manufacturer's protocol, respectively. Overexpression or knockdown of UCA1 were confirmed by qRT-PCR, knockdown of EZH2 were confirmed by qRT-PCR and western blot analysis.

### RNA Extraction and qRT-PCR (quantitative RT‑PCR (qRT‑PCR)

Total RNA was extracted using TRIzol reagent (Invitrogen) and cDNA synthesis was performed using a reverse transcription kit (Promega, Madison, WI, USA) according to the manufacturer's instructions. For qRT-PCR, the mRNA level of UCA1 was measured using the SYBR Green PCR Master Mix (Applied Biosystems, Waltham, MA, USA) and the Applied Biosystems 7900HT sequence detection system (Applied Biosystems). EZH2 mRNA relative expression level was evaluated using the 2^-ΔΔCt^ method and normalized to glyceraldehyde 3-phosphate dehydrogenase (GAPDH). Primers sequences were listed in supplementary Table S1.

### Cell Counting Kit-8 (CCK-8) Assay

MKN45 and MGC803 cells in logarithmic growth phase were collected and seeded in 96-well plates at a density of 3×10^4^/well. After cell culture for 24 h, 100 μL cisplatin (Selleck, Houston, USA) with different concentrations of 0, 5, 10, 15, 20 and 25 µM were added to each well, respectively. The blank wells were set up for calibration. There were 6 replicates for each group. After incubation for another 24 h, a total of 10 µL of CCK-8 (Dojindo, Kumamoto, Japan) reagent were added to the wells and the cells were allowed for 2 h of reaction, OD value was measured at 450 nm by spectrophotometry (BioTek, Vermont, USA).

### Apoptosis Assay

Transfected GC cells were treated with 10 µM cisplatin. After 24 hours of culture, both attached and the floating cells were harvested, washed twice with ice-cold PBS, and suspended in 100 µl binding buffer. The cells were stained with 3 µl Annexin V-FITC and 5 µl propidium-iodide (PI) and incubated at room temperature for 15 min in the dark. Finally, 300 µl 1× binding buffer was added to each sample of cells. Apoptosis was analyzed by flow cytometry using the FACSCalibur system (BD Biosciences, USA).

### Western blot analysis

The proteins were extracted from cell samples and western blot was performed as previously described 19. The antibodies involved were purchased from Cell Signaling Technology.

### RNA immunoprecipitation (RIP)

RIP assays were performed by using the Magna RIP RNA-Binding Protein Immunoprecipitation Kit (Millipore, USA) according to the manufacturer's instructions. Briefly, cells were lysed in lysis buffer, and cleared lysates were immunoprecipitated with the indicated anti-EZH2 and anti-IgG antibodies (Cell Signaling Technology, USA). Immunoprecipitated and input RNA were isolated and reverse transcribed for following qRT-PCR amplifications with UCA1 specific primers. The primers used for amplification are listed in Table 1.

### RNA pull-down

RNA pull-down assays were performed using Magnetic RNAProtein Pull-Down Kit (Pierce, Rockford, IL, USA) according to the manual. Briefly, RiboMAX Large Scale RNA Production Systems (Promega) were used to yield full length of UCA1. The UCA1 RNA was bound to the beads to orient the RNA for protein binding after biotin labeling. RNA-bound beads were added into cell protein lysate for immunoprecipitation. The beads were washed and the samples were eluted with SDS-PAGE Loading Buffer for western blot analysis.

### Statistical analysis

All experimental results were repeated at least three times and are shown as mean ± standard deviation (s.d.). Differences between treated and control groups were analyzed using the Student's t-test and one-way ANOVA. A two-tailed value of P < 0.05 was considered statistically significant. All statistical analyses were performed with the Stata software 12.0 (Stata Corporation, College Station, TX, USA).

## Results

### UCA1 highly expressed in human GC tissues and associated with poor prognosis

**Figure 1:**
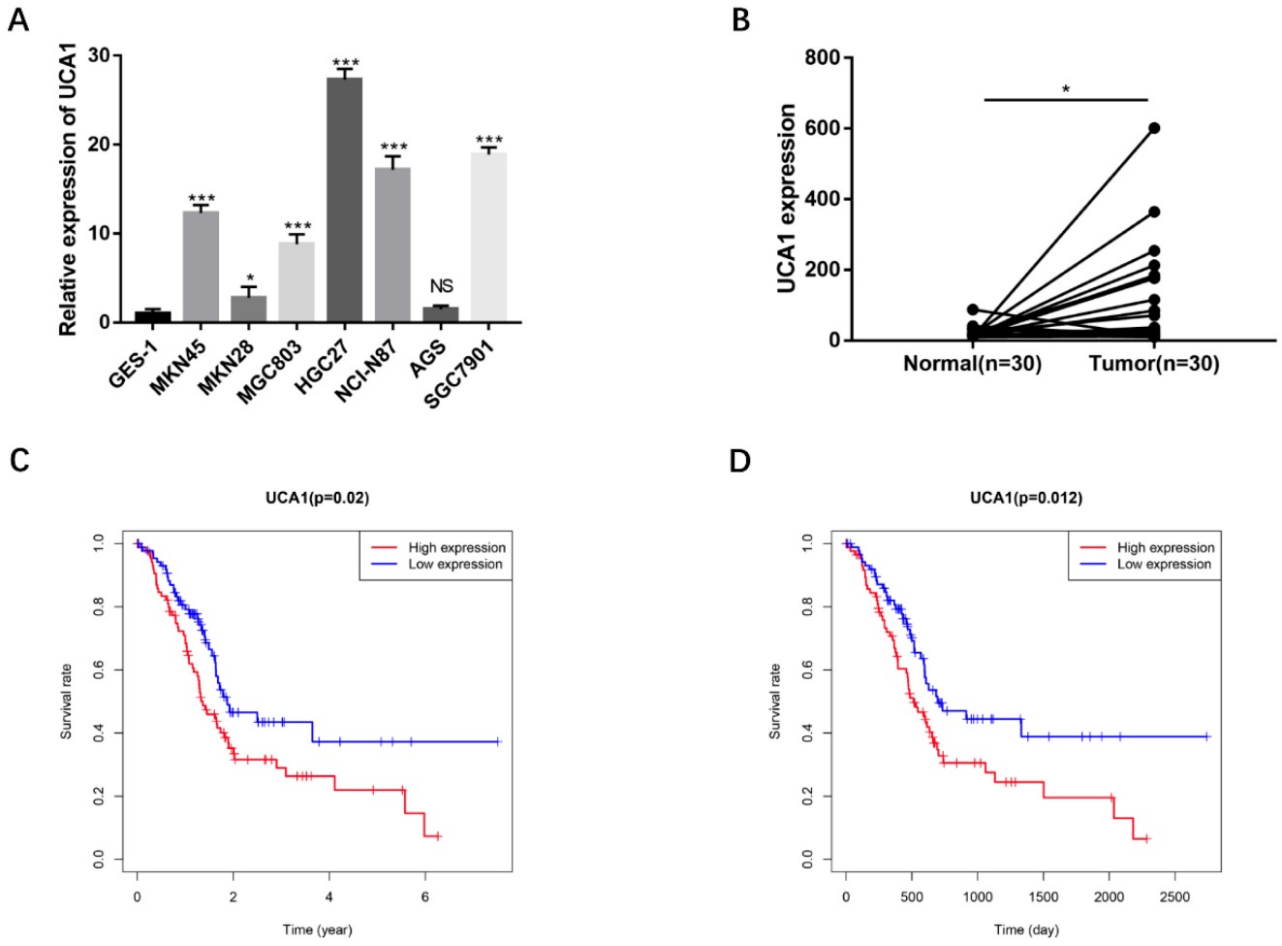
** UCA1 overexpresses in GC cells and GC tissues, correlates with poor survival. A** UCA1 expression in GC cell lines including MKN45, MKN28, MGC803, HGC27, NCI-N87and AGS. The expression of UCA1 was normalized to that in GES-1. **B** The expression of UCA1 in 30 GC tissues and matched normal samples were detected using quantitative reverse transcription PCR. **C** The association of expression level of UCA1 with TCGA GC patients' survival. D The association of expression level of UCA1 with GEO GC patients' survival. *P < 0.05, ***P < 0.001, 'NS' means not significant.

To validate the biological functions of UCA1 in GC progression, we investigated the expression levels of UCA1 in GC cell lines by qRT-PCR and found that major GC cell lines presented markedly higher expression than GES-1 (Figure 1A). To confirm this result, we detected UCA1 expression in tumor tissues and their matched nontumor tissues of 30 patients with GC by qRT-PCR and found that UCA1 expression level was significantly higher in tumor tissues than that in nontumor tissues (Figure 1B). Moreover, we analyzed the relationship between the expression level of UCA1 in GC tissues and patients' clinicopathological characteristics based on TCGA database. The expression level of UCA1 in tumor tissues was positively associated with lymph node metastasis (P = 0.004), distance metastasis (P = 0.015**)** and higher TNM stage (P = 0.035), but not with other clinicopathological parameters including age, gender and T stage (Table 1). We further validated these results using the GEO(GSE62254) database which showed that the expression level of UCA1 in GC tissues was also significantly associated with lymph node metastasis (P = 0.009), distance metastasis (P = 0.005) and higher TNM stage (P = 0.024) of GC (Table 2). Besides, the expression levels of UCA1 in GC tissues significantly correlated with the poor survival of GC patients from TCGA database (P = 0.02, Figure 1C), this result was further validated by GEO cohort (P=0.012, Figure 1D), indicating UCA1 might participate in the development of GC.

### Over-expression of UCA1 promotes GC cell proliferation and inhibits cisplatin-induced apoptosis

**Figure 2:**
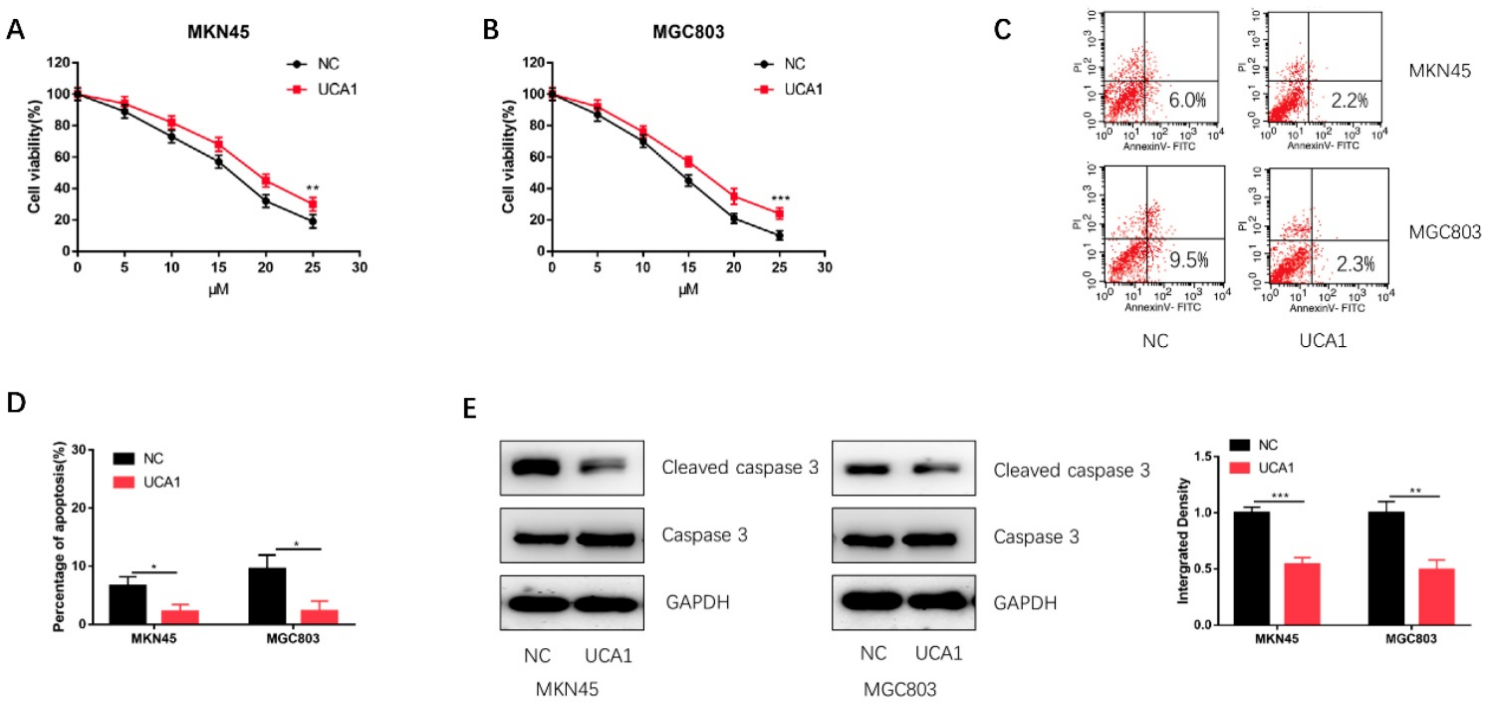
** Overexpression of UCA1 promoted proliferation and inhibited cisplatin-induced apoptosis. A** Overexpression of UCA1 promoted the proliferation of MKN45 cells treated with different cisplatin concentrations. **B** Overexpression of UCA1 promoted the proliferation of MGC803 cells treated with different cisplatin concentrations. **C** Over-expressed UCA1 inhibited apoptosis of MKN45 and MGC803 cells induced by 10 µM cisplatin for 24 h. **D** Over-expressed UCA1 inhibited cleaved caspase 3 expression in MKN45 and MGC803 cells induced by 10 µM cisplatin for 24 h. *p < 0.05, **p < 0.01, ***p < 0.001.

To investigate the role of UCA1 in the cisplatin treatment of GC, we performed CCK-8 assay to detect the effect of different concentrations of cisplatin on the proliferation of GC cells. We found that the proliferation capacities of MKN45 and MGC803 cells were markedly enhanced after UCA1 overexpression in a concentration-dependent manner (Figure 2A and 2B). Additionally, UCA1 overexpression decreased the apoptosis of MKN45 and MGC803 cells treated with 10 µM of cisplatin for 24 h (Figure 2C and 2D). Western blot analysis also suggested that the expression of cleaved caspase-3 was downregulated after UCA1 was overexpressed (Figure 2E). These results indicated that UCA1 could promote GC cell cisplatin-resistance via reducing cisplatin-induced apoptosis.

### Knockdown of UCA1 decreases GC cell proliferation and promotes cisplatin-induced apoptosis

**Figure 3:**
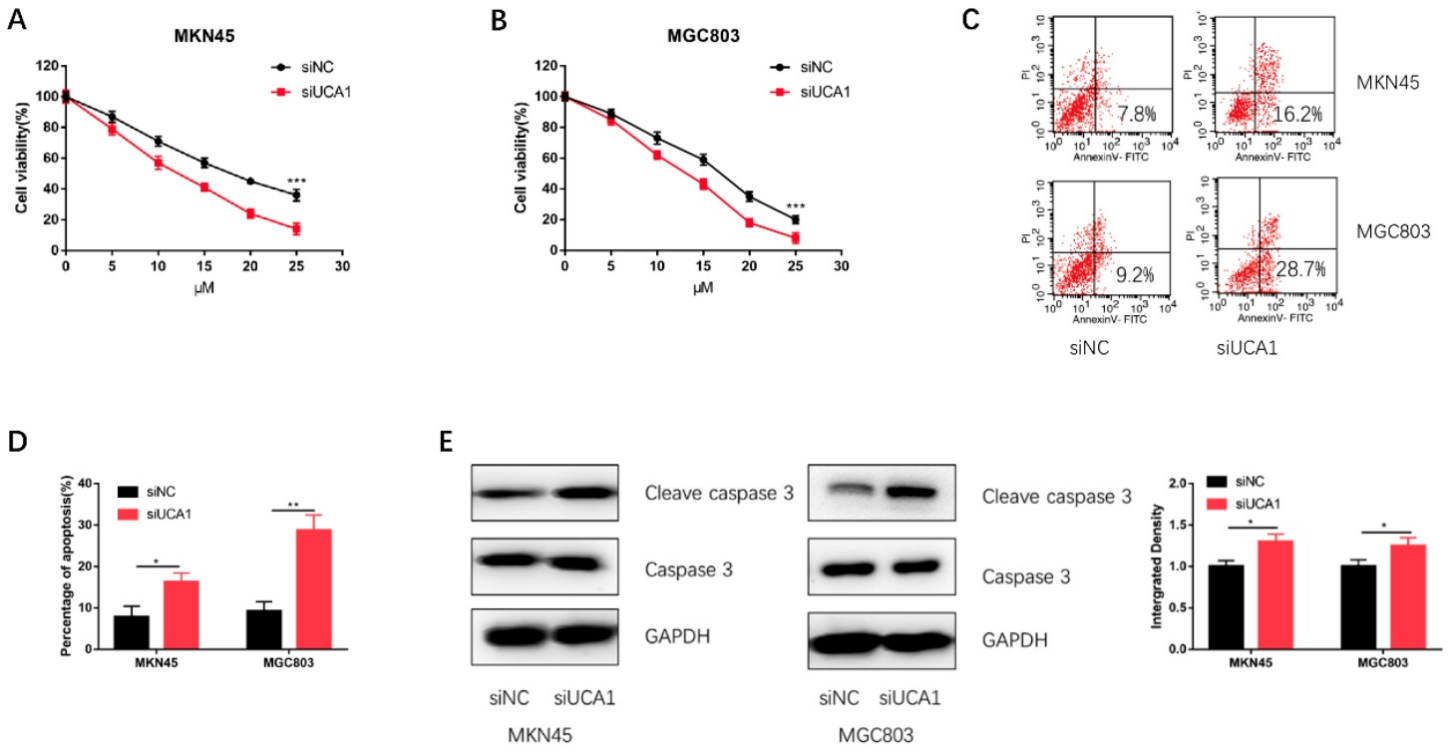
** Knockdown of UCA1 decreased GC cell proliferation and promoted cisplatin-induced apoptosis. A** Downregulated UCA1 inhibited proliferation of MKN45 cells treated with different cisplatin concentrations. **B** Downregulated UCA1 inhibited proliferation of MGC803 cells treated with different cisplatin concentrations. **C** Downregulated UCA1 promoted apoptosis of MKN45 and MGC803 cells induced by 10 µM cisplatin for 24 h. **D** Downregulated UCA1 promoted cleaved caspase 3 expression in MKN45 and MGC803 cells induced by 10 µM cisplatin for 24 h. *p < 0.05, **p < 0.01, ***p < 0.001.

To further validate UCA1 could promote GC cell cisplatin resistance, we specifically knockdown UCA1 using siRNA and then performed CCK-8 assay and apoptosis assay on MKN45 and MGC803 cells. We discovered that knockdown of UCA1 significantly decreased the proliferation capacities of MKN45 and MGC803 cells (Figure 3A and 3B). To further confirm this result, we performed apoptosis assay by flow cytometry. The apoptosis assay demonstrated UCA1 knockdown increased the apoptosis of MKN45 and MGC803 cells treated with 10 µM cisplatin for 24 h (Figure 3C and 3D). After UCA1 was knockdown, apoptosis-related protein expressions of GC cells were also detected. Western blot analysis showed that the expression of cleaved caspase-3 was upregulated after UCA1 was down-regulated (Figure 3E). These results, taken together, indicated that UCA1 contributes to GC cell cisplatin resistance via inhibiting cisplatin-induced apoptosis.

### UCA1 regulates EZH2 expression and knockdown of EZH2 promotes cisplatin-induced apoptosis in GC cells

**Figure 4:**
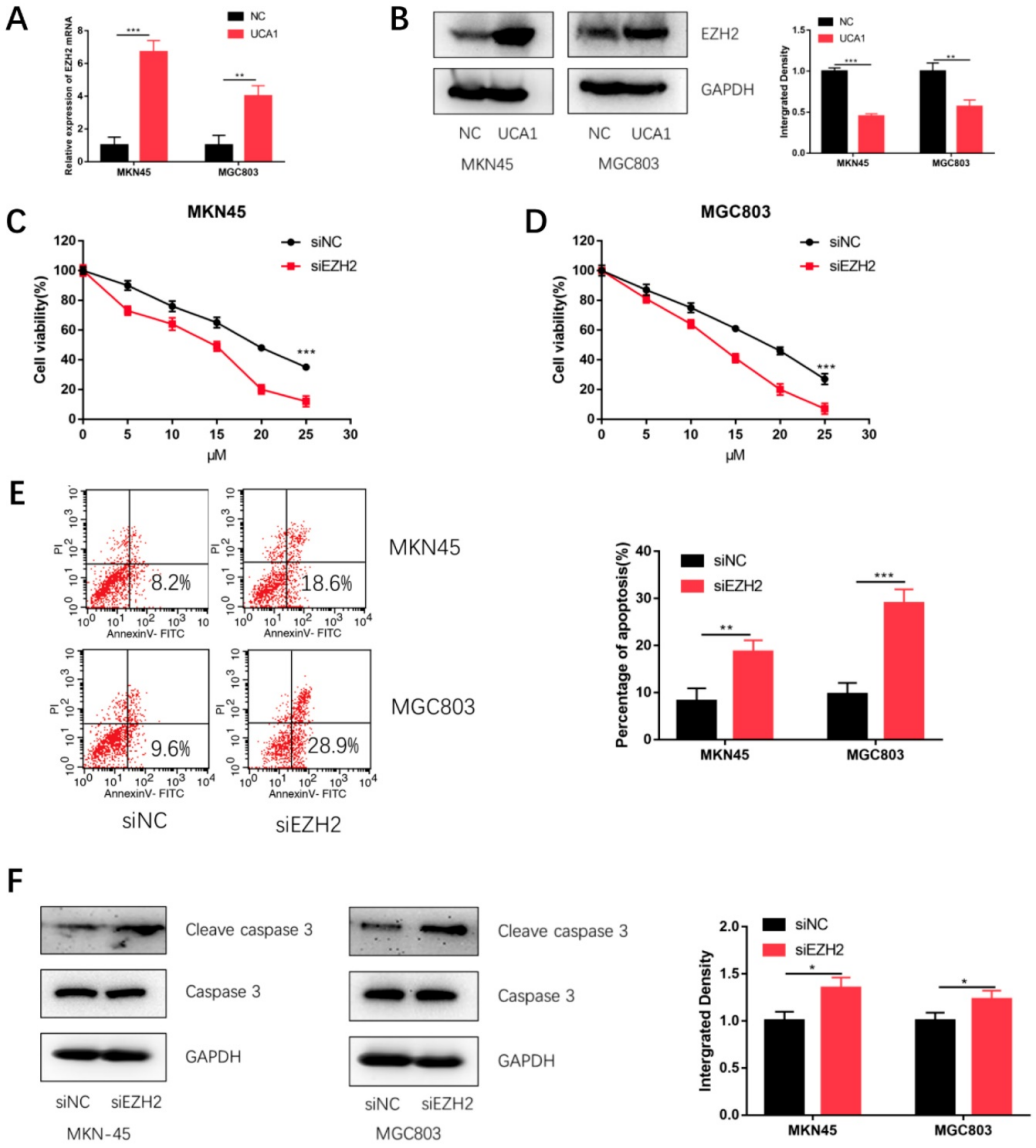
** UCA1 regulates EZH2 expression, knockdown of EZH2 decreases GC cell proliferation and promotes cisplatin-induced apoptosis in GC cells. A** Over-expression of UCA1 promoted mRNA expression of EZH2. **B** Western blot analysis was used to detect the protein level of EZH2 after UCA1 overexpression. **C** Downregulated EZH2 inhibited MKN45 cells proliferation in different cisplatin concentrations. **D** Downregulated EZH2 inhibited MGC803 cells proliferation in different cisplatin concentrations. **E** Downregulated EZH2 promoted apoptosis of MKN45 and MGC803 cells induced by 10 µM cisplatin for 24 h. **F** Downregulated EZH2 promoted cleaved caspase 3 expression in MKN45 and MGC803 cells induced by 10 µM cisplatin for 24 h. *p < 0.05, **p < 0.01, ***p < 0.001.

Based on our previous studies that UCA1 regulated the levels of EZH2 in GC^18^, we postulated that UCA1 might elevate the protein levels of EZH2 to promote the cisplatin resistance of GC. We firstly performed qRT-PCR assays, which showed that the EZH2 mRNA expression was significantly increased after UCA1 was over expressed (Figure 4A). To further validate this result, we performed western blot assay with the similar result (Figure 4B). These results together suggested that UCA1 upregulates the protein levels of EZH2.

To testify whether EZH2 could promote GC cisplatin resistance, we specifically knockdown EZH2 using siRNA and then performed CCK-8 assay and apoptosis assay. The efficiency of si-EZH2 was validated by both qRT-PCR (Figure S1A) and western blot (Figure S1B). MKN45 and MGC803 cells were then treated with different concentrations of cisplatin after EZH2 knockdown. CCK-8 assay showed that the proliferation of MKN45 and MGC803 cells were also attenuated (Figure 4C and 4D). Moreover, EZH2 knockdown increased cisplatin-induced apoptosis and cleaved caspase-3 expression (Figure 4E and 4F). These results indicated knockdown EZH2 could decrease GC cell proliferation and enhance cisplatin-induced apoptosis in GC cells.

### UCA1 promotes cisplatin resistance of GC cells via activating PI3K/AKT pathway

**Figure 5:**
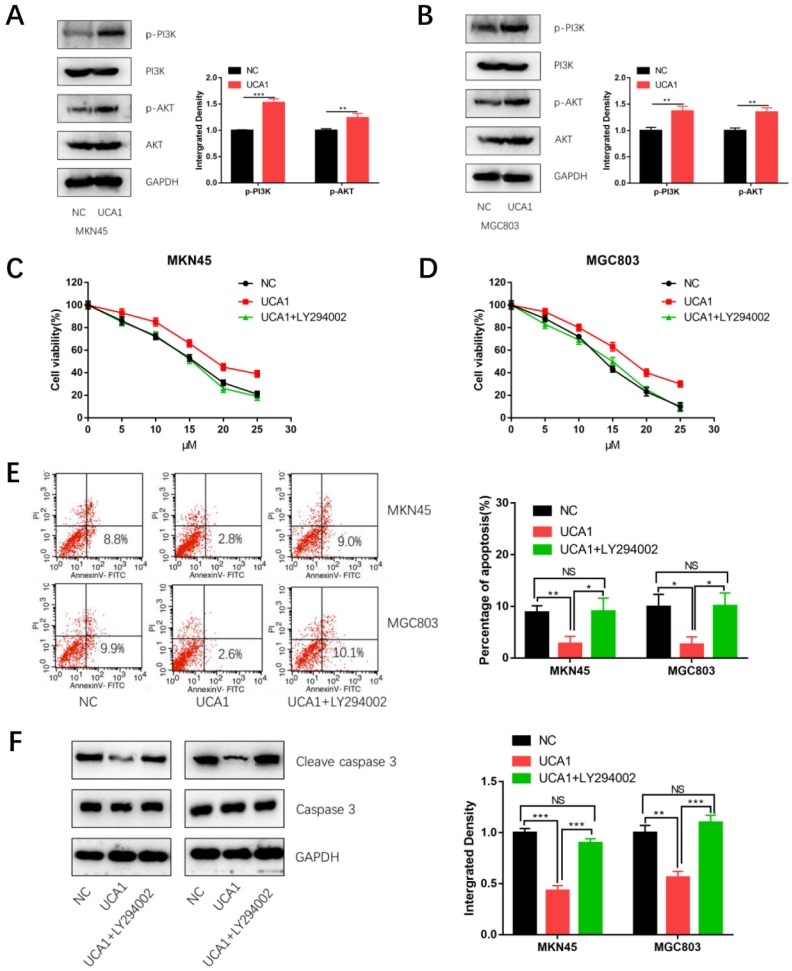
** UCA1 promotes cisplatin resistance of GC cells via activating PI3K/AKT pathway. A, B** Western blot analysis was performed to detect the expression of PI3K/AKT pathway after overexpression of UCA1 in MKN45 and MGC803 cells. **C, D** The CCK8 assay showed that in the presence of LY294002, the increased proliferation ability of MKN45/UCA1 and MGC803/UCA1 cells was impaired. **E** The apoptosis assay, which indicated that in the presence of LY294002, the UCA1-induced cisplatin resistance ability of GC cells was diminished. **F** Western blot analysis demonstrated that the downregulation of cleave caspase 3 due to the overexpression of UCA1 was attenuated. *p < 0.05, **p < 0.01, ***p < 0.001, 'NS' means not significant.

To explore the mechanism by which UCA1 regulates cisplatin resistance of GC, we detected the proteins level of phosphorylation-PI3K (p-PI3K), total PI3K, phosphorylation-AKT (p-AKT) and total AKT respectively using western blot analysis. As shown in Figure 5A and 5B, expression of p- PI3K and p-AKT were significantly higher in the UCA1 overexpression cells than that in negative control cells. This result showed over-expressed UCA1 activated PI3K/AKT pathway in GC cells. To further testify whether UCA1 promotes cisplatin resistance of GC cells via activating PI3K/AKT pathway, the cells were pretreated with LY294002 (a specific inhibitor of PI3K/AKT). The CCK8 assay showed that in the presence of LY294002, the increased proliferation ability of MKN45/UCA1 and MGC803/UCA1 cells was impaired (Figure 5C and 5D). To further confirm this result, we performed the apoptosis assay, which indicated that in the presence of LY294002, the UCA1-induced cisplatin resistance ability of GC cells was diminished (Figure 5E). Moreover, the downregulation of cleave caspase 3 due to the overexpression of UCA1 was also attenuated (Figure 5F). These results indicated that UCA1 increases cisplatin resistance of GC via activation of the PI3K/AKT pathway.

### UCA1 elevates EZH2 to activate the PI3K/AKT pathway

**Figure 6:**
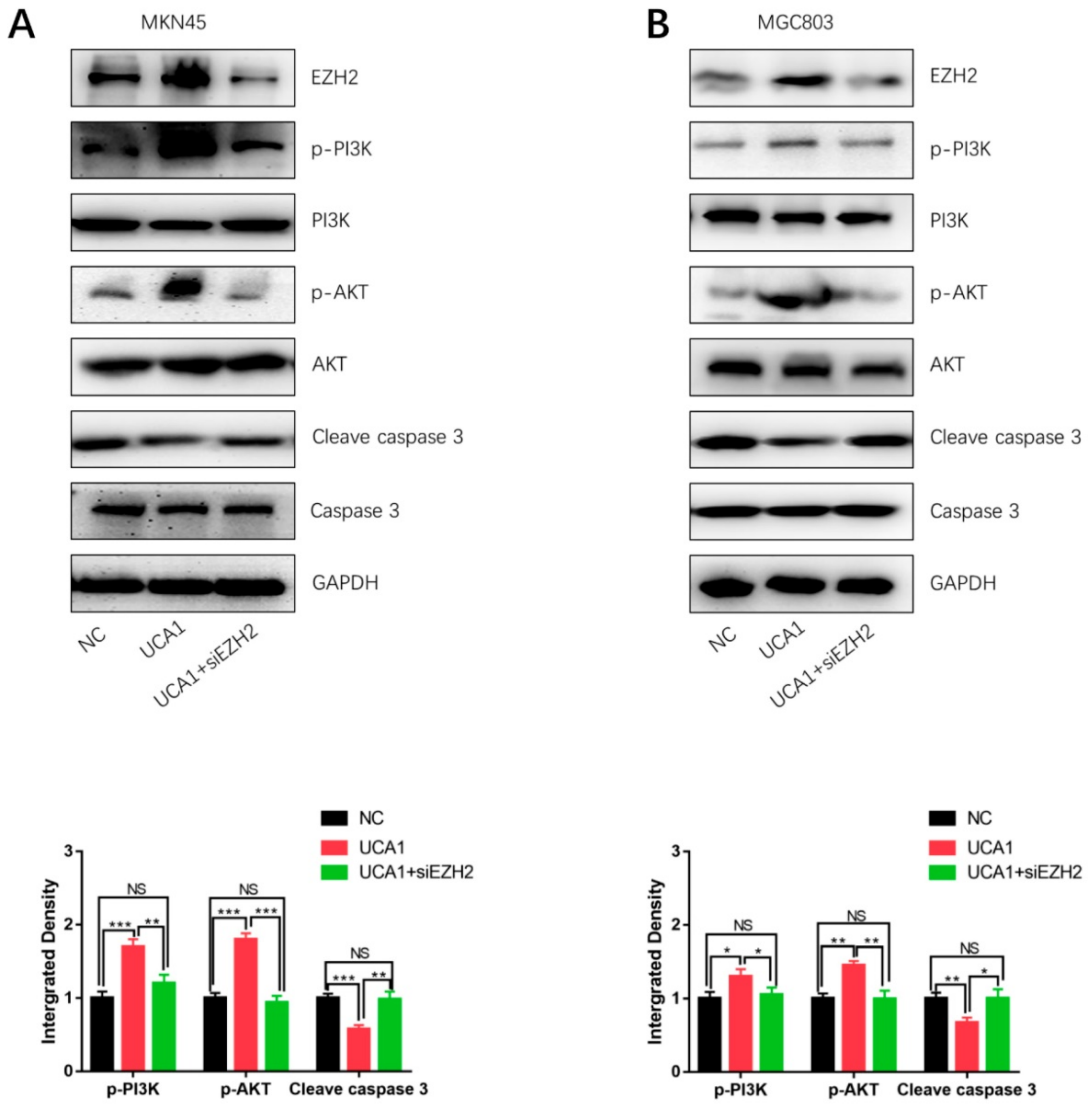
** UCA1 regulates EZH2 to activate the PI3K/AKT pathway. A, B** Western blot analysis showed that knockdown of EZH2 could significantly decreased the expression of p-PI3K and p-AKT, which were induced by the overexpression of UCA1. *p < 0.05, **p < 0.01, ***p < 0.001, 'NS' means not significant.

In order to determine how UCA1 activates the PI3K/AKT pathway in cisplatin resistance of GC, we specifically knockdown EZH2 in MKN45/UCA1 and MGC803/UCA1 cells. As shown in Figure 5A and 5B, western blot analysis showed that inhibition of EZH2 could markedly decreased the expression of p-PI3K and p-AKT, which were induced by the overexpression of UCA1. On the other hand, after knockdown of EZH2, the downregulation of cleave caspase 3 due to the overexpression of UCA1 was impaired (Figure 6A and 6B). Taken together, these results indicated that UCA1 activated the PI3K/AKT pathway mainly through upregulating EZH2.

### UCA1 directly interact with EZH2 in gastric cancer cell

**Figure 7:**
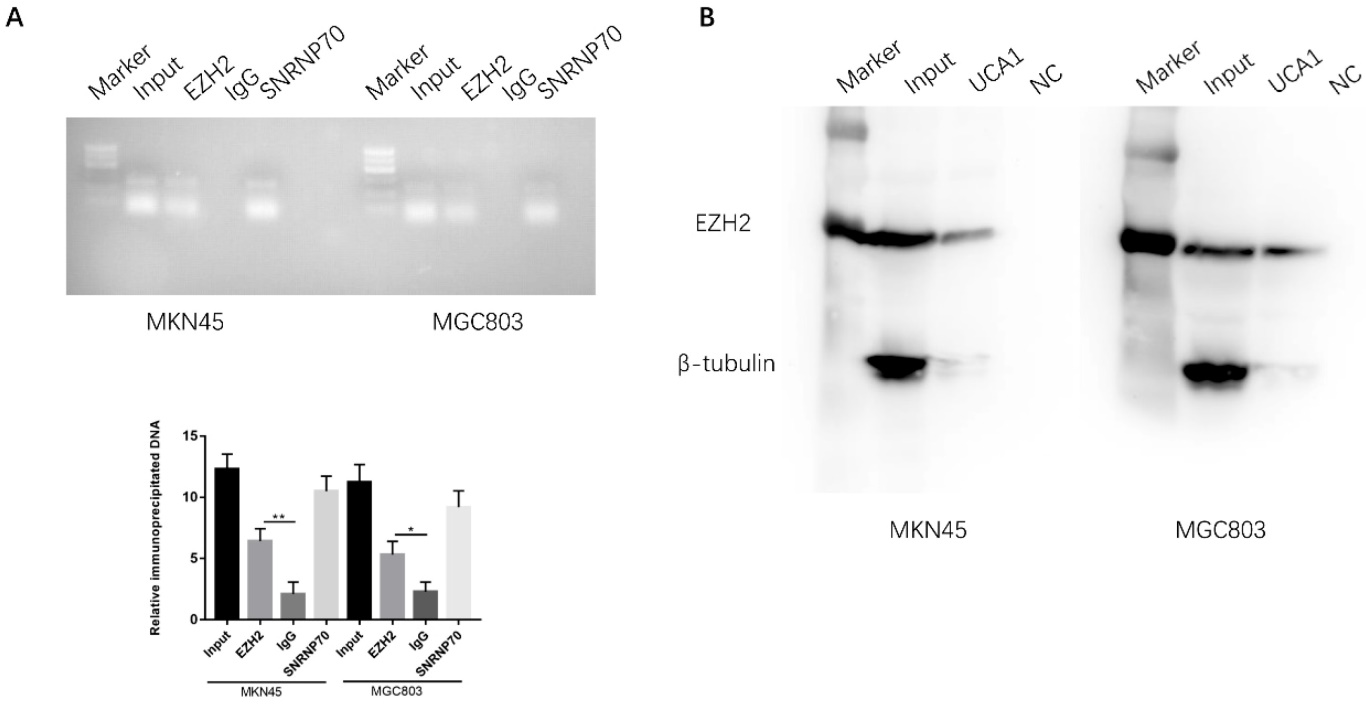
** UCA1 interacts with EZH2 in GC. A** RIP assay was performed to determine the association between UCA1 and EZH2. Anti-EZH2 significantly harbored more UCA1 fragments than anti-IgG via RT-PCR. Anti-SNRNP70 was used as a positive control. B RNA pull-down assay was used to further confirm the interaction between UCA1 and EZH2. UCA1 RNA-bound EZH2 protein was detected via western blot and no band showed up in negative control. β-actin acted as the loading control. *p < 0.05, **p < 0.01.

EZH2 is a crucial component of PRC2, which was reported physically associated with one-fifth of lncRNAs to date 20, 21. We postulated that UCA1 may interact with and bind to EZH2 to regulate downstream molecular events in view of the regulation of UCA1 on EZH2 protein expression. To determine whether the interaction between UCA1 and EZH2, we performed RNA immunoprecipitation assay (RIP). RIP assay showed that UCA1 was significantly enriched with the EZH2 antibody compared with IgG in MKN45 and MGC803 cells, IgG was used as negative control and SNRNP70 was used as positive control (Figure 7A). This result indicated that UCA1 could interact with EZH2. To further confirm the assumption, RNA pull-down assay was performed. We found that biotin-labeled UCA1 could harbor EZH2 protein. And β-tubulin protein was not detected after biotin-labeled UCA1 precipitation, which suggested UCA1 could specially interact with EZH2 protein (Figures 7B). These results showed that UCA1 could directly interact with EZH2.

## Discussion

Drug resistance of cancer cells is one of the major causes of chemotherapy failure 22. LncRNAs are reported to induce the drug resistance by regulating drug metabolism, autophagy, apoptosis and epithelial-mesenchymal transition 23-26. We previously elucidated that lncRNA UCA1 promotes tumor development and metastasis in GC 18, 27. He et al. 14 demonstrated that lncRNA UCA1 predicts a poor prognosis and regulates cell proliferation and migration in GC. Besides, UCA1 has been reported to promotes cisplatin resistance in Ovarian Cancer 13, non-small-cell lung cancer^12^, oral squamous cell carcinoma 11 and bladder cancer 15, including GC 10. Furthermore, it is reported that knockdown of UCA1 significantly promoted apoptosis by regulating Bax and cleaved caspase-3/9 expressions 8. When apoptosis occurs, the activated pro-apoptotic protein promotes the release of pro-apoptotic substances from the mitochondria into the cytoplasm 7. This process causes caspase-dependent programmed cell death (apoptosis) in tumor cells. Enhancer of Zeste Homologue 2 (EZH2) has a crucial role in gene expression regulation, which has been reported to contributed to the cisplatin resistance of GC 28, 29. Besides, researches showed that overexpression of EZH2 could mainly activate PI3K/AKT pathway in tumor progression 18, 30. Many researches have shown that the PI3K/AKT pathway plays a very important role in chemotherapy resistance 30. Inactivation of PI3K/AKT pathway can reverse the drug resistance, thus recovering the sensitivity of tumor cells to chemotherapeutic drugs 30. Recently research showed that the PI3K/AKT pathway mainly affects the chemotherapy resistance through multiple drug-resistance related-proteins and anti-apoptosis proteins 31, 32. Chemotherapeutic drugs in tumor cells induce apoptosis through various ways to achieve the anti-tumor effect. PI3K/AKT signaling pathway is one of major pathways regulating apoptosis, which inhibits expressions of caspase-9 and caspase-3 31, 32.

Here in this study, we found that UCA1 was significantly higher in GC tissues compared with matched nontumor tissues. And overexpression of UCA1 is associated with high lymph node metastasis rates, high distance metastasis rates and late TNM stage indicating UCA1 has an oncogenic role in GC. Higher UCA1 in GC tissues significantly correlated with the poorer survival of GC patients, indicating UCA1 could act as a prognostic factor of GC. In this study, we also found that down-regulation of UCA1 increased the sensitivity of CC cells to cisplatin and promoted cell apoptosis. Our previous study suggested that UCA1 regulated the levels of EZH2 in GC 18. Over-expressed UCA1 in MKN45 and MGC803 cells significantly increased the mRNA expression of EZH2. Furthermore, EZH2 knockdown in MKN45 and MGC803 cells showed the same cellular functions as UCA1 overexpression. In order to further explore the mechanism, we examined the expressions of key proteins in PI3K/AKT signaling pathway. Results demonstrated that p-PI3K and p-AKT were markedly upregulated after overexpression of UCA1. Furtherly, our results suggested that knockdown EZH2 could retort the UCA1-induced upregulation of p-PI3K and p-AKT. In addition, one-fifth of all human lncRNAs identified is physically associated with EZH2 20, 21. To determine the association between UCA1 and EZH2, RIP and RNA pull down assay were performed. The RIP assay result showed that EHZ2 protein could bind to UCA1 RNA, which reflected the direct interaction between EZH2 and UCA1. Then, RNA pull down assay demonstrated that EZH2-specific harbored by biotin labeled UCA1 RNA. These results suggested that the direct interaction between EZH2 and UCA1. All above results indicated that UCA1 could play a role in regulating cisplatin resistance in GC through recruiting EZH2 and activating PI3K/AKT pathway to modulate cell apoptosis. Clearly, EZH2 has multiple highly complex roles in the regulation of PI3K/AKT pathway and can positively or negatively regulate other pathways. The overall impact of EZH2 levels on PI3K/AKT pathway is not straightforward and may vary on different cell types, thus the impact of EZH2 remains to be clarified. Even so, treatments targeting UCA1 or EZH2 might provide meaningful therapeutic strategies for cisplatin resistance GC patients.

## Conclusion

In conclusion, we found that the lncRNA UCA1 promoted the cisplatin resistance of GC via recruiting EZH2 and activating PI3K/AKT pathway to modulate cell apoptosis.

These findings further define the importance of lncRNAs in tumor drug resistance and suggest that UCA1 or EHZ2 provide meaningful therapeutic strategies for cisplatin resistance GC patients.

## Supplementary Material

Supplementary figure.

## Figures and Tables

**Table 1:** Association of UCA1 expression with clinicopathologic characteristics of gastric cancer based on TCGA database.

	UCA1 expression			
Parameters	Cases	Low(n=221)	High(n=103)	p-value((χ2-test)
Age(years)				
<60	101	65	36	0.316
≥60	223	156	67	
Gender				
Male	201	139	62	0.641
Female	123	82	41	
T stage				
T1+T2	85	62	23	0.275
T3+T4	239	159	80	
Lymph node metastasis				
Negative	122	95	27	0.004^**^
Positive	202	126	76	
Distance metastasis				
Negative	158	118	40	0.015^*^
Positive	166	103	63	
TNM stage				
I + II	178	132	46	0.035^*^
III + IV	156	99	57	

*P<0.05, **P<0.01.

**Table 2:** Association of UCA1 expression with clinicopathologic characteristics of gastric cancer based on GEO (GSE62254) database.

	UCA1 expression			
Parameters	Cases	Low(n=142)	High(n=101)	p-value((χ2-test)
Age(years)				
<60	83	50	33	0.681
≥60	160	92	68	
Gender				
Male	166	97	69	0.999
Female	77	45	32	
T stage				
T1+T2	148	81	67	0.143
T3+T4	95	61	34	
Lymph node metastasis				
Negative	59	43	16	0.009^**^
Positive	184	99	85	
Distance metastasis				
Negative	170	109	61	0.005^**^
Positive	72	32	40	
TNM stage				
I + II	102	68	34	0.024^*^
III + IV	140	73	67	

## Abbreviations

UCA1: urothelial cancer associated 1
GC: gastric cancer
EZH2: Enhancer of Zeste Homologue 2
