# Supplementary material for: LncRNA UCA1 promotes cisplatin resistance in gastric cancer via recruiting EZH2 and activating PI3K/AKT pathway
Source: J Cancer. 2020 Apr 6;11(13):3882–92. doi: 10.7150/jca.43446 (PMC7171500; doi:10.7150/jca.43446)
Supplement: Supplementary file 1 — Supplementary figure. [file jcav11p3882s1.pdf]

Figure S1:

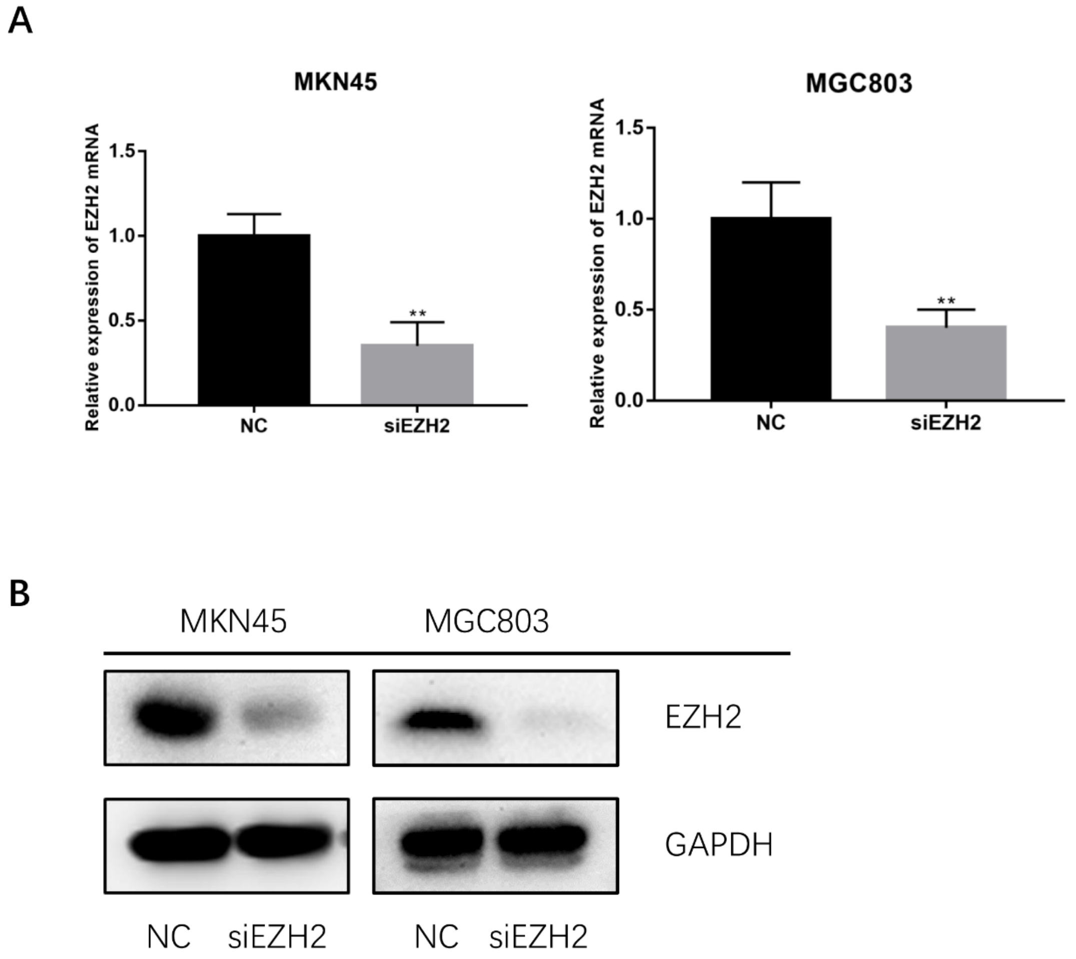

Figure S1. The knockdown efficiency of siEZH2 A The knockdown efficiency of siEZH2 on MKN45 and MGC803 cells was detected by qRT-PCR. **B** Western blot analysis was used to detect the knockdown efficiency of siEZH2 on MKN45 and MGC803 cells.
